# Induction of somatic embryogenesis and cryopreservation of *Abies pinsapo* Boiss.

**DOI:** 10.3389/fpls.2025.1535113

**Published:** 2025-01-29

**Authors:** Laura Cabero-Moreno, Ignacio Landeras-López, María Victoria Bravo-Navas, Carolina Sánchez-Romero

**Affiliations:** Departamento de Botánica y Fisiología Vegetal, Universidad de Málaga, Málaga, Spain

**Keywords:** biotechnology, cryoconservation, embryogenic cultures, slow freezing, spanish fir

## Abstract

*Abies pinsapo* is an endangered species, endemic to southern Spain. Somatic embryogenesis and cryopreservation constitute important biotechnological tools, which can be used in order to improve the management and conservation of threatened species. The objective of this work was to develop somatic embryogenesis and cryopreservation protocols for *A. pinsapo*. Somatic embryogenesis was induced from mature zygotic embryos of *A. pinsapo* cultured on solid MS medium with macroelements at half-strength and supplemented with 20 g L^-1^ sucrose and 5 mg L^-1^ 6-benzylaminopurine (BA). Embryogenic cultures successfully proliferated on solid medium with the same formulation supplemented with 20 g L^-1^ sucrose, 500 mg L^-1^ L-glutamine, 1 g L^-1^ casein hydrolysate and 1 mg L^-1^ BA. Different preconditioning and cryoprotective treatments were tested in order to optimize cryopreservation of embryogenic tissues by using the slow-cooling technique. Embryogenic cultures at their exponential growth phase, i.e. 12-14 days after the last subculture, were used as cryopreservation explants. The best results were achieved after sucrose preculture and cryoprotecion with PGD I (mixture of polyethylene glycol, glucose and DMSO I), with 100% of explants resuming somatic embryogenesis after thawing. Following fluorescein diacetate (FDA) staining, more intense and abundant green fluorescence could be observed in these samples, compared to those subjected to other preconditioning and cryoprotective treatments, thus evincing a higher proportion of viable cells after freezing in liquid nitrogen. Cold hardening did not improve cryotolerance. In fact, incubation at 5 °C for two weeks appeared to affect explants response, delaying tissue regrowth after cryopreservation. This is the first time in which somatic embryogenesis and cryopreservation have been reported in Spanish fir. The results obtained allow to establish the bases for the integration of these techniques into *in situ* and *ex situ* conservation strategies.

## 1 Introduction

The Spanish fir (*Abies pinsapo* Boiss.) is a species endemic to southern Spain. Its distribution area is very limited, with three main localizations in the Sierra de las Nieves, Sierra de Grazalema, and Sierra Bermeja (Alba-Sánchez et al., 2019).

Different factors threat *A. pinsapo* populations, such as the fire, agriculture, grazing, timber harvesting, insects, like *Dioryctria aulloi*, *Mindarus abietinus, Cryphalus numidicus*, *Cinara confinis* and *Cinara pectinatae*, and fungi, like *Heterobasidion annosum* and *Armillaria mellea* (Esteban et al., 2010). Although evidences of demographic genetic erosion among cohorts have not been found; bottlenecks, genetic drift, and a low among-populations genetic differentiation have been reported in this species (Cobo-Simón, 2020). Moreover, increased drought and warming induced by climate change can lead to a decrease of its ecological niche, thus worsening its future prospects (Gutierrez Hernández et al., 2017). These circumstances make *A. pinsapo* a highly vulnerable species and currently it is listed in different regional, national and international conservation catalogues, including the International Union for Conservation of Nature’s Red List of Threatened Species, where it is classified as endangered under criteria B1ab(i,ii,iii)+2ab(i,ii,iii) (IUCN, 2024).

Spanish fir stands, relict tree formations descended from ancient tertiary forests, are also protected, being included in the EU Habitats Directive (Council Directive 92/43/EEC on the conservation of natural habitats and of wild fauna and flora) and in the Natura 2000 Network (Gómez-Zotano et al., 2023).

In conifers, different strategies have been utilized for the management and conservation of genetic resources, including the application of biotechnological tools, such as somatic embryogenesis and cryopreservation.

Somatic embryogenesis consists of the development of embryos from somatic cells. This *in vitro* culture technique presents multiple applications, some of which can be used for the restoration and conservation of threatened species (Sánchez-Romero, 2019, 2021). In fact, it has been applied for conservation purposes in different endangered forest trees, such as *Castanea dentata*, *Tsuga canadensis*, *Tsuga caroliniana*, *Chamaecyparis thyoides*, *Fraxinus pennsylvanica*, *Fraxinus americana* (Merkle et al., 2017), and *Abies nebrodensis* (Jouini et al., 2023). Somatic embryogenesis is an efficient regeneration system, with a high multiplication capacity (Salaj et al., 2011), which can be exploited for the rapid propagation of high value genotypes for reforestation (Gupta et al., 1993). Moreover, somatic embryogenesis can be used as a complement in breeding programs aiming the generation of genotypes resistant to the main stressors threatening the species, as the possibility of propagating trees vegetatively provides significant advantages for capturing and enhancing genetic gain in plant improvement (Lambardi et al., 2018). Besides, as an *in vitro* culture technique capable of regenerating a whole plant from a single cell, somatic embryogenesis allows the application of biotechnological tools, such as genetic transformation, somaclonal variation, somatic hybridization, *in vitro* mutagenesis, production of haploid and double-haploid plants, and *in vitro* selection, greatly increasing plant breeding results. An important attribute of embryogenic cultures is their amenability to cryopreservation (Lambardi et al., 2018). Cryopreservation, i.e., storage at –196°C, the temperature of liquid nitrogen, is considered the best method for the long-term conservation of plant genetic resources (Engelmann, 2011). It requires a small space and limited maintenance, and decreases the risk of culture loss by contamination or human error (Engelmann, 2004; Winkelmann et al., 2004). Furthermore, cryopreservation allows the integration of somatic embryogenesis within practical forestry (Carson, 1986), as it avoids the occurrence of somaclonal variation and loss of regeneration capacity in cultures (Lambardi et al., 2008), and contributes to maintain the juvenility of donor tissues (Krajňáková et al., 2013).

The combined use of somatic embryogenesis and cryopreservation improves the management and conservation of interesting genotypes. Moreover, they are significant components of advanced breeding programs (Cyr, 1999), considerably enhancing the ability to obtain superior genotypes (Martínez et al., 2003), and contributing to preserve conventional bred material or biotechnological products until the results of field and progeny testing are available (Krajňáková et al., 2013).

Although somatic embryogenesis and cryopreservation constitute very valuable biotechnological tools, which can be incorporated to *in situ* and *ex situ* conservation strategies, they have not been reported in Spanish fir. The objective of this work was to develop somatic embryogenesis and cryopreservation protocols for *A. pinsapo* conservation. For this purpose, the slow-cooling technique was applied, as it is the cryopreservation method most commonly used for storage in liquid nitrogen of conifer embryogenic masses (Häggman et al., 2000; Ballesteros et al., 2024). As indicated by Engelmann (Engelmann, 2011), the steps to carry out the slow-freezing protocols are preconditioning of tissues, cryoprotection, slow cooling to a temperature close to -40°C, rapid immersion in liquid nitrogen, and thawing. In the present investigation, different treatments related to the preconditioning and cryoprotection steps were tested. Growth dynamics of *A. pinsapo* embryogenic cultures was also investigated as the culture growth phase at which explants are sampled has a critical effect on their subsequent cryopreservation response (Bradaï et al., 2017), due to its influence on tissue cell composition and physiological state. In general, in conifer embryogenic cultures, higher cryotolerance has been reported with tissues taken during the exponential or early-exponential growth phases (Salaj et al., 2010, 2022). At this stage, cells have small vacuoles and reduced water content, which improves freezing survival (Reinhoud et al., 2000). Active and vigorous growth observed at these phases is also considered decisive to withstand cryopreservation (Häggman et al., 2000; Salaj et al., 2010).

## 2 Materials and methods

### 2.1 Plant material

Mature cones of Spanish fir (*Abies pinsapo* Boiss.) were harvested from open-pollinated trees growing in Sierra de Yunquera (36°43′51″N 4°57′11″W), in the Sierra de las Nieves National Park (Malaga, Spain), following the indications of the Spanish fir recovery plan (Consejería de Agricultura, Ganadería, Pesca y Desarrollo Sostenible, Junta de Andalucía, Spain) (Spanish Fir Recovery Plan, 2024). Collected cones were stored at 5-7 °C in darkness until further use.

### 2.2 Initiation and proliferation of embryogenic cultures

Mature embryos excised from mature cones were used as explants for somatic embryogenesis initiation. For disinfection, cones were sequentially washed with detergent, rinsed with running tap water, agitated for 1 min in 70% (v/v) ethanol and rinsed twice in distilled water. Then, seeds were carefully removed from scales and dewinged, and surface sterilized for 20 min in a 0.5% (v/v) sodium hypochlorite solution supplemented with Tween 20 (10 drops L^-1^), followed by three rinses in sterile distilled water for 5 min each. Sterilized seeds were cut lengthwise under aseptic conditions and the mature zygotic embryos carefully excised and horizontally inoculated on culture media. Induction medium consisted of the Murashige and Skoog (MS) formulation (Murashige and Skoog, 1962), with macroelements at half-strength, supplemented with 20 g L^-1^ sucrose, and solidified with 3 g L^-1^ Phytagel™ (Sigma). For initiation of somatic embryogenesis different concentrations of 6-benzylaminopurine (BA) were tested (0.1, 0.5, 1 and 5 mg L^-1^). Twenty-eight to seventy embryos were utilized per treatment. Cultures were incubated in the dark at 25 ± 1°C and examined periodically.

Putatively embryogenic calli were transferred to proliferation medium, consisting of MS medium, with macroelements at half-strength, supplemented with 20 g L^-1^ sucrose, 500 mg L^-1^ L-glutamine, 1 g L^-1^ casein hydrolysate and 1 mg L^-1^ BA, and gelled with 3 g L^-1^ Phytagel™ (Sigma). Cultures maintenance was executed by repetitive subcultures onto fresh medium at three-four week intervals. Proliferating cultures were incubated at 25 ± 1 °C in darkness.

The pH of all media was adjusted to 5.74 before adding the gelling agent. Media sterilization was carried out by autoclaving for 20 min at 121°C and 0.1 MPa.

### 2.3 Cultures growth kinetics

In order to use optimal tissues as cryopreservation explants, growth dynamics of Spanish fir embryogenic cultures was investigated. For this purpose, 0.3 g of embryogenic callus were cultured on proliferation medium and maintained under standard maintenance conditions for six weeks. Fresh weight increase was monitored 4 days after culture initiation and weekly thereafter. Twenty replicates were used.

### 2.4 Cryopreservation

**Figure 1:**
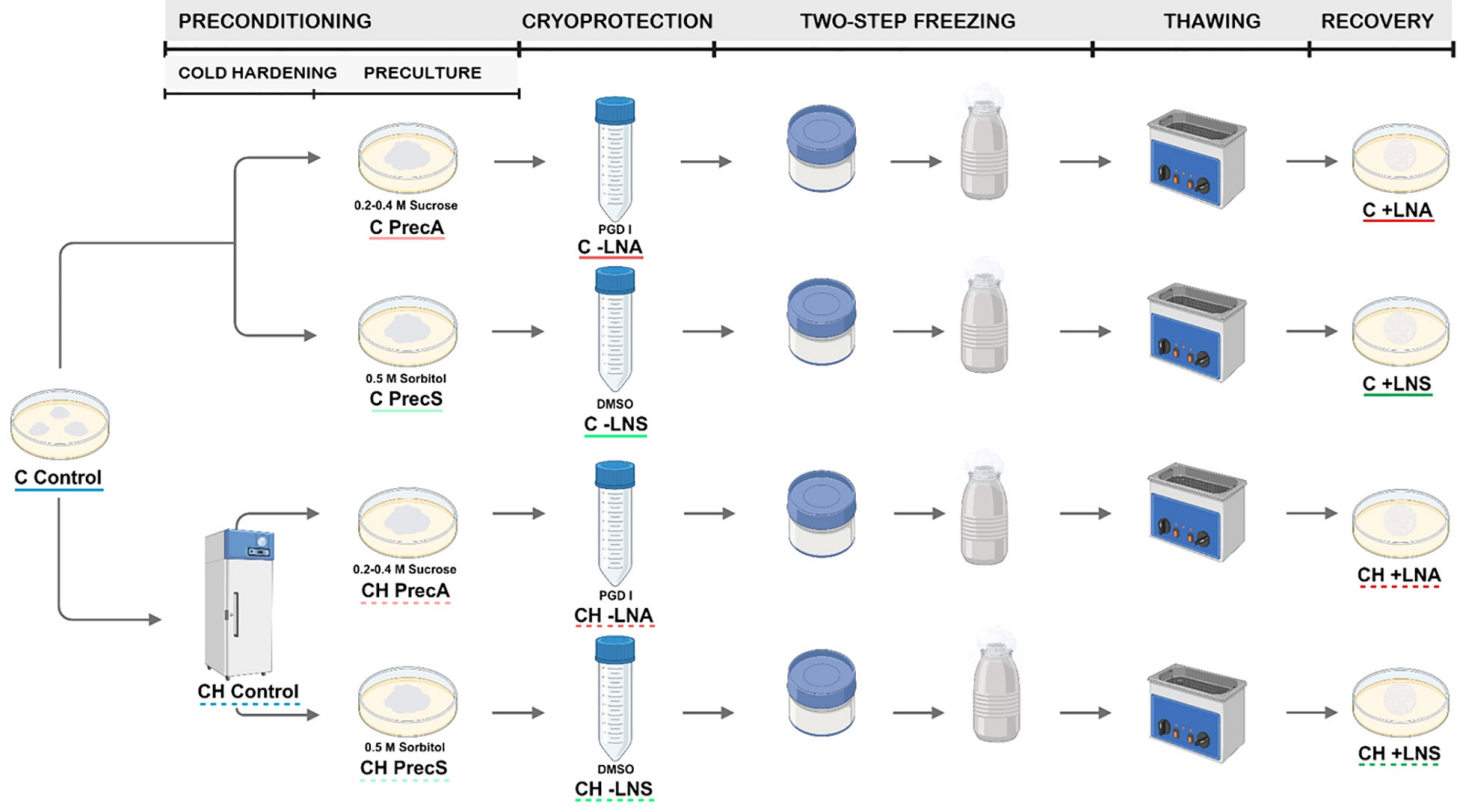
Cryopreservation of embryogenic cultures of *A. pinsapo* by using the slow-freezing technique. Scheme showing the steps of this procedure (preconditioning, cryoprotection, two-step freezing, thawing and recovery) and the different preconditioning and cryoprotective treatments tested following the protocols A and S. Control: samples directly collected from stock cultures, untreated (C Control) or after incubation at 5°C for two weeks. (CH Control). Prec: preculture controls, consisting of samples precultured but not subjected to cryoprotection and freezing, with (CH Prec) or without (C Prec) a previous cold hardening treatment. -LN: treated controls, consisting of samples precultured and cryoprotected, but not frozen, with (CH -LN) or without (C -LN) a previous cold hardening treatment. +LN: samples subjected to the complete cryopreservation protocol, with (CH +LN) or without (C +LN) a previous cold hardening treatment.

Cryopreservation experiments were carried out using embryogenic cultures of Spanish fir initiated from mature zygotic embryos and maintained as pointed above. Different options related to the preconditioning and the cryoprotection steps were tested using the slow-cooling method (**Figure 1**).

Based on the investigations of Salaj et al. (2010, 2016, 2020, 2022) in *A. alba* x *A. cephalonica*, *A. alba* x *A. numidica*, and *A. alba*, the effects of a preconditioning treatment with sorbitol and dimethylsulfoxide (DMSO) cryoprotection were tested using a modified protocol named S. According to previous experiences applying the two-step freezing technique to embryogenic cultures of other tree species, such as avocado (Guzmán-García et al., 2013) and olive (Sánchez-Romero et al., 2009), a higher final DMSO concentration was used. For cryopreservation, embryogenic tissue selected from stock cultures 12-14 days after the last subculture were cultured onto solidified proliferation medium supplemented with 0.5 M sorbitol. After incubation for 24 h, 3 g embryogenic calli were transferred to 50 ml polystyrene tubes containing 9 ml of basal proliferation medium supplemented with 0.5 M sorbitol. Subsequently, liquid basal proliferation medium with 0.5 M sorbitol and 15% (v/v) DMSO was gradually added to the tubes placed on ice over a period time of 30 min up to a final concentration of 7.5% (v/v) DMSO. After incubation for 30 min, 1.8 ml of the cell suspension was transferred to a 2 ml-cryovial. The cryovials were quickly inserted into “Mr Frosty™” freezing containers (Thermo Scientific™) filled with isopropanol, and transferred to an ultra-freezer (-80°C) to achieve a cooling rate of approximately -1°C min^-1^. When the temperature into the cryotubes reached -40°C, they were immediately plunged into liquid nitrogen for at least 1 h. Thawing was carried out into a sterile water bath at 40°C until the ice had melted. Samples were then dispersed onto sterile filter paper discs to remove the liquid and the upper ones were placed onto proliferation medium. One day later, filter papers with embryogenic cells on top were transferred to new proliferation medium.

The sucrose preculture and cryoprotective solution utilized by Aronen et al. (1999) for cryopreservation of *A. cephalonica* were in parallel tested using a modified protocol named A. In order to achieve comparable results, the protocol of Aronen et al. (1999) was modified by using the same tissue amounts and solution volumes indicated above. In this case, prior to cryopreservation, embryogenic calli were precultured in proliferation medium supplemented with 0.2 M sucrose for 24 h, and with 0.4 M sucrose for an additional 24 h. Afterwards, 3 g embryogenic tissue were transferred to 50 ml polystyrene tubes containing 9 ml of basal proliferation medium supplemented with 0.4 M sucrose. Subsequently, 9 ml of the PGD I solution, consisting of 10% (w/v) polyethylene glycol 6000, 10% (w/v) glucose and 10% (v/v) DMSO dissolved in distilled water, were progressively added to the tubes placed on ice. After incubation for 30 min, subsequent freezing and rewarming steps were executed as previously indicated.

The effect of a previous cold-hardening treatment was also tested. For this purpose, 12-14 days after the last subculture, embryogenic masses were incubated in proliferation medium at 5 °C in the dark for 14 days. Subsequently, embryogenic calli were cryopreserved as described above, testing the same pretreatments with osmotic agents and cryoprotectants (**Figure 1**).

**Figure 2:**
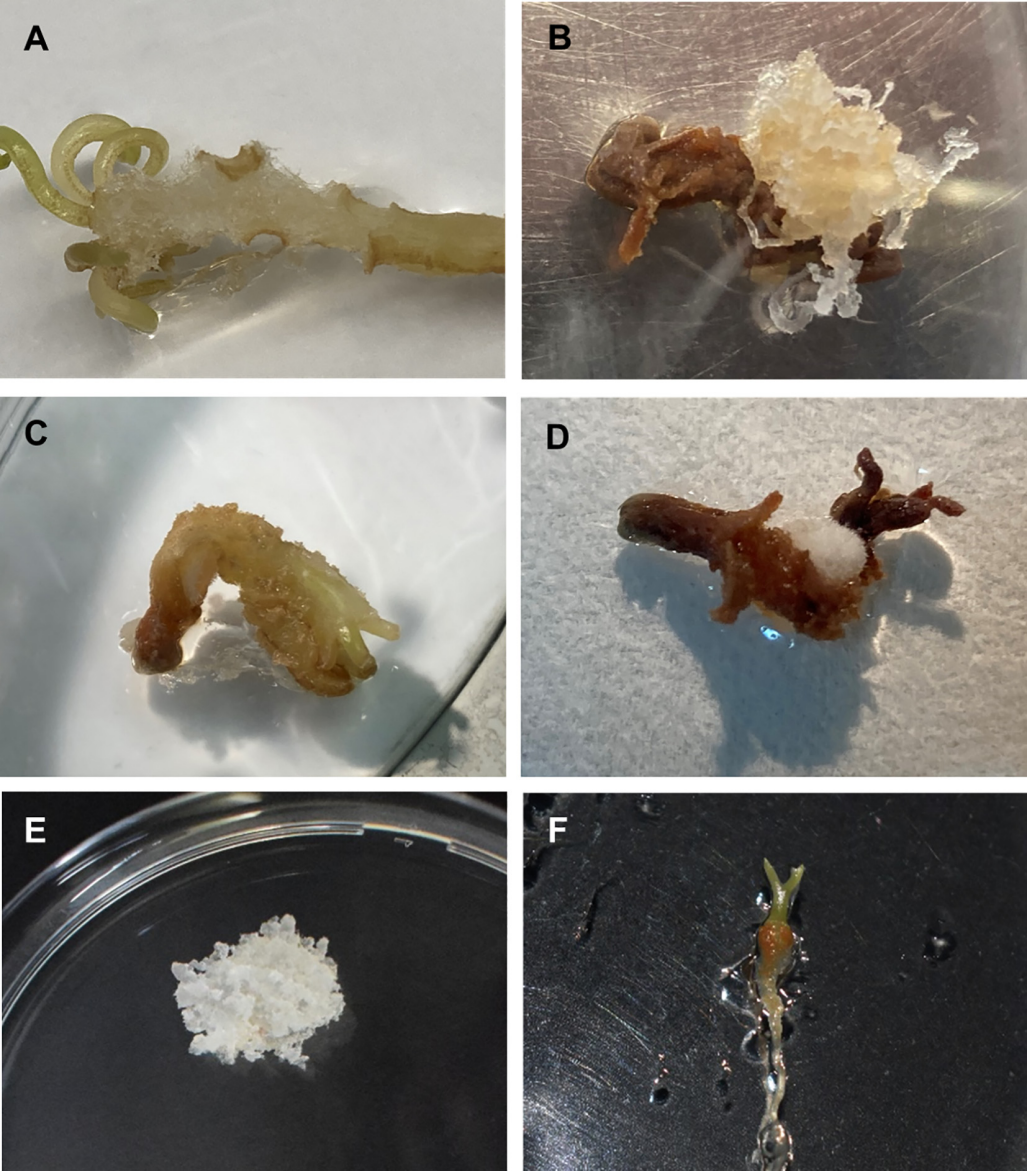
Induction of somatic embryogenesis from mature zygotic embryos of *A*. *pinsapo*. **(A)** Callus arising from the hypocotyl-cotyledon region of a zygotic embryo. Initiation of different types of callus from zygotic embryos: **(B)** white translucent and mucilaginous mass, **(C)** soft, beige to light-brown callus, and **(D)** compact white mass with superficial elongated filaments. **(E)** Embryogenic tissue growing on proliferation medium. **(F)** Plantlet developed from embryogenic cultures of Spanish fir.

Five-ten replicates were performed per treatment and the experiment was repeated at least twice. Data of survival, recovery and culture appearance were monitored during three successive recultures of three weeks each, three, six and nine weeks after thawing. Survival was assessed as the percentage of samples showing any sign of regrowth, while recovery was evaluated as the percentage of explants in which regrown tissue exhibited embryogenic features (**Figure 2E**). To examine the effect of the different cryopreservation steps, distinct controls were included: untreated controls (control), consisting of samples directly collected from stock cultures; preculture controls (Prec), consisting of samples precultured but not subjected to cryoprotection and freezing; and treated controls (-LN), consisting of samples precultured and cryoprotected, but not frozen. In order to analyze the effect of cold-hardening, additional controls were included: cold-hardened controls (CH control), consisting of samples directly collected from stock cultures and incubated at 5°C for two weeks, but without any additional preculture or cryopreservation treatment, and the corresponding controls of the different preconditioning and cryoprotective treatments tested (CH Prec and CH -LN).

### 2.4.1 Effect of preconditioning treatment on callus proliferation rate

Due to the influence of growth conditions of embryogenic tissues on their subsequent response to cryopreservation, the proliferation rate of cultures subjected to the different preconditioning treatments was evaluated. For this purpose, 0.3 g of embryogenic callus derived from the different treatments tested were transferred to proliferation medium and incubated at 25 ± 1 °C in darkness. Fresh weight increase was determined after a culture cycle, three weeks later. At least ten cultures were included per treatment.

### 2.4.2 Fluorescein diacetate staining

Cell viability was analyzed by using the fluorescein diacetate (FDA) assay, following the protocol of Widholm (1972). For tissue staining, several drops of a 0.02% (w/v) FDA solution was directly added to the samples. After 4 min of incubation at room temperature, green fluorescence was observed under a stereo microscope Multizoom AZ-100 (Nikon, Tokyo, Japan), with an epifluorescence system CoolLED’s pE-300, filter GFP-B (EX 460–500; DM 505; BA 510–560) and zoom lens (Nikon AZ Plan APO 1x).

Three weeks after cryopreservation, at least three samples from independent cultures were stained per treatment. Photographs were taken with a Nikon DS-5Mc digital cooled camera (Nikon, Tokyo, Japan).

### 2.5 Statistical analysis

Percentage data were subjected to frequency analysis with an R×C test of independence or with a three-way log-linear analysis, using the BIOMstat software (Exeter Software, Setauket, NY, USA). The rest of data were analyzed by ANOVA and significant differences among treatments were determined by the Tukey´s HSD test, using the software package SPSS 28.0 (IBM SPSS Statistics, Armonk, NY, USA). The significance level used was 0.05 in all cases (Sokal and Rohlf, 2013).

## 3 Results

### 3.1 Initiation and proliferation of embryogenic cultures

First signs of callus initiation could be observed two weeks after explants culture on induction medium. Although disorganized tissue started to develop in different parts of the initially cultured zygotic embryos, it mostly occurred on the hypocotyl region (**Figure 2A**). Different types of calli could be distinguished: white translucent and mucilaginous masses, exhibiting embryogenic features (**Figure 2B**), beige to light-brown, friable and soft calli (**Figure 2C**), and compact, white masses with superficial elongated filaments (**Figure 2D**). As time in culture progressed, formed calli evolved in different ways. Beige and soft tissue masses normally turned brown and died. Despite white, compact calli with translucent elongated cells increased in size, noticeable changes in their general appearance were not evident and clear embryogenic structures could not be identified. Callus tissues initially identified as putatively embryogenic could continue growing, giving rise to the establishment of embryogenic cultures (**Figure 2E**), but growth cessation and modification of the developmental pattern were also observed.

High percentages of callus formation were obtained in all the BA concentrations tested (**Table 1**). However, embryogenic cultures only were established from mature embryos incubated in culture medium supplemented with 5 mg L^-1^ BA. Embryogenic cultures of *A. pinsapo* successfully grew on proliferation medium, exhibiting sustained and rapid growth and maintenance of the embryogenic features. In the absence of a cytokinin supplement, occasional development of somatic embryos could be observed, as well as plantlet formation with well-developed shoot and root poles (**Figure 2F**).

**Table 1:** Effect of BA concentration on total and embryogenic callus induction from mature zygotic embryos of *A. pinsapo*.

BA concentration (mg L^-1^)	Total callus (%)	Embryogenic callus (%)
0.1	62.12	0
0.5	69.23	0
1	77.78	0
5	80.00	4

### 3.2 Cultures growth kinetics

**Figure 3:**
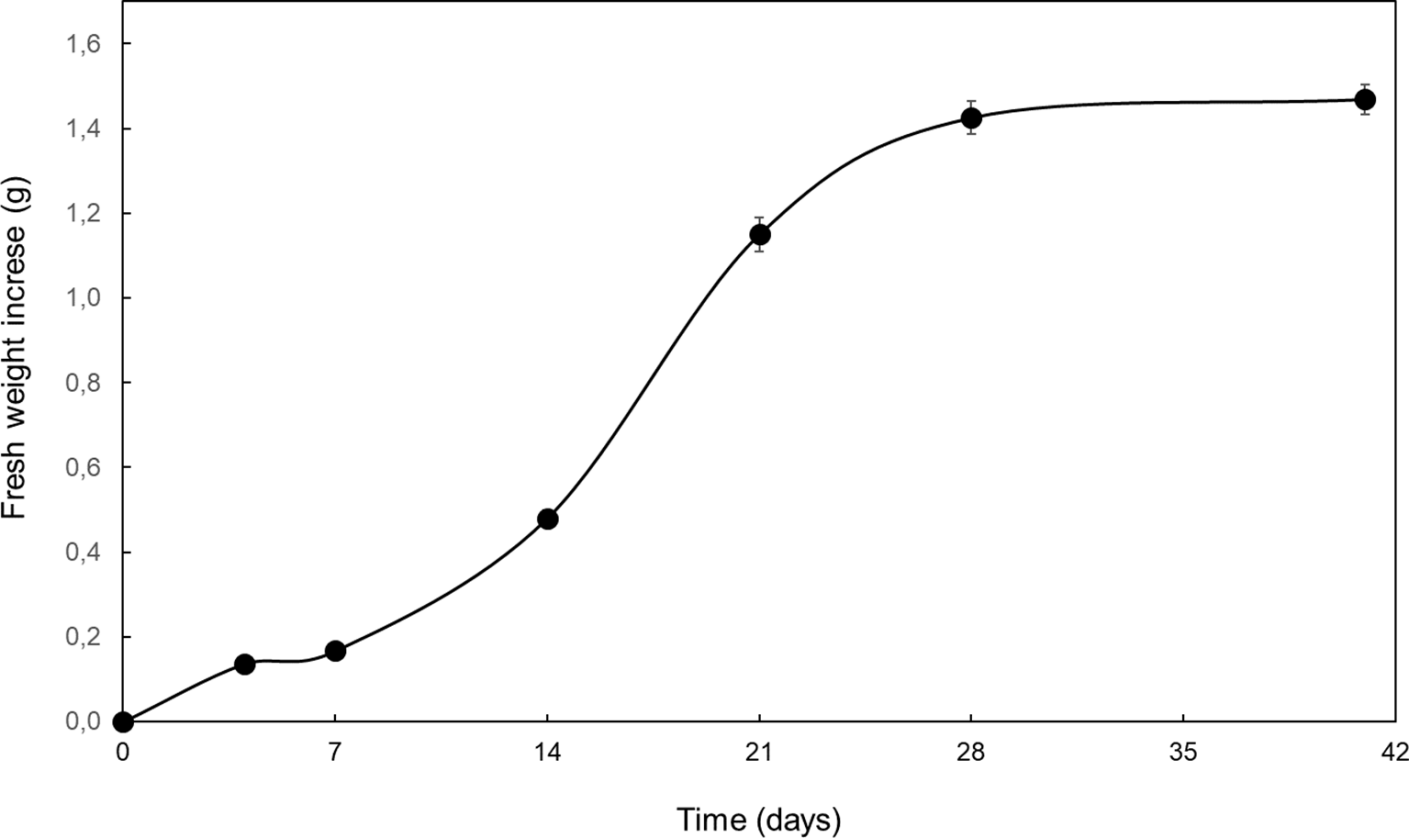
Fresh weight increase of embryogenic cultures of *A. pinsapo* cultured in proliferation medium throughout a 6-week culture period. Data presented are mean ± SEM.

Growth of Spanish fir embryogenic cultures under proliferation conditions displayed a typical sigmoidal curve (**Figure 3**), in which different phases could be distinguished: lag (0-7 d), exponential (8-14 d), lineal (15–21 d) deceleration (22-28 d) and stationary (29-42 d) (Bohjwani and Dantu, 2013).

### 3.3 Cryopreservation

Survival and recovery data were coincident, as in all cases new tissues growing after cryopresevation exhibited embryogenic features.

**Figure 4:**
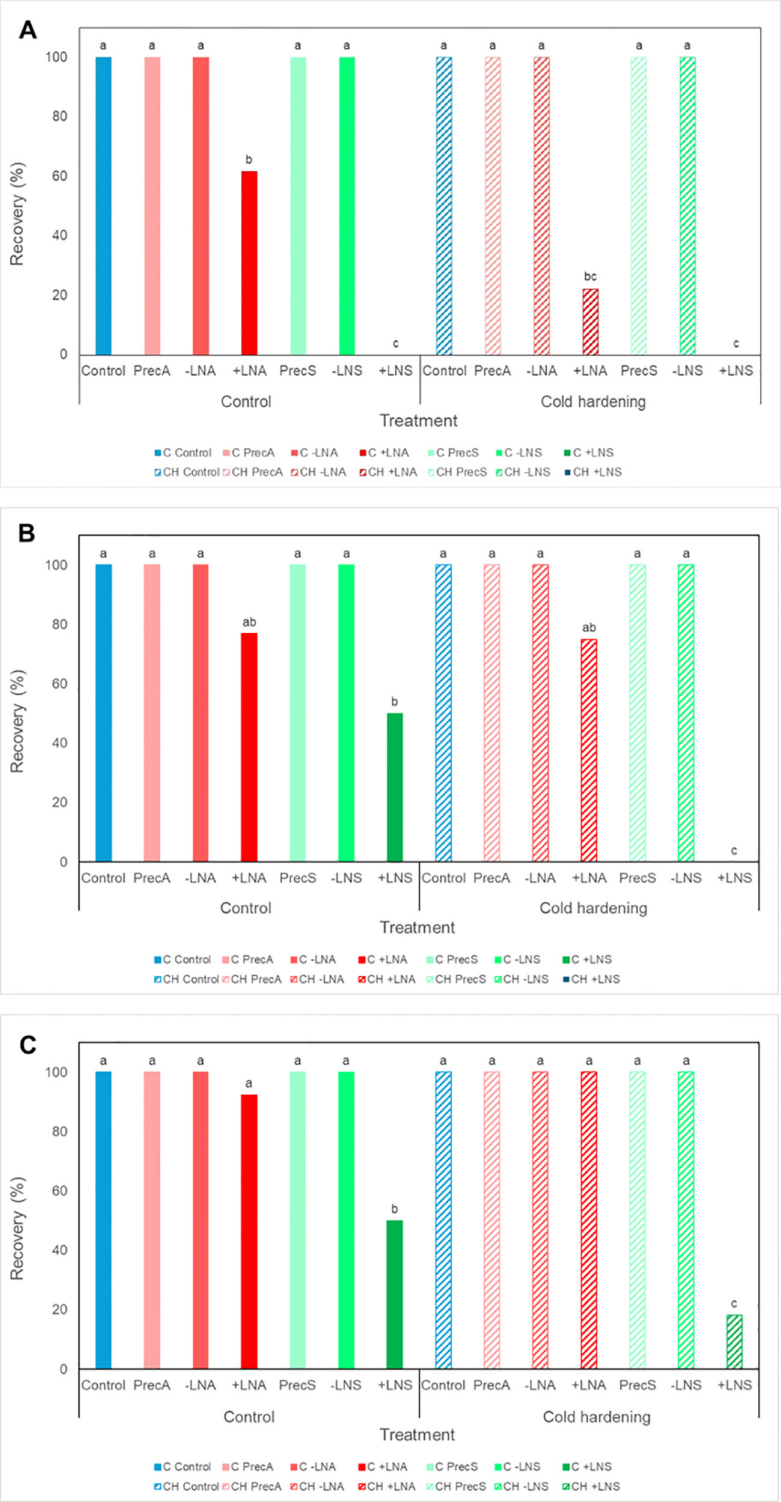
Recovery rates of embryogenic cultures of *A*. *pinsapo* untreated (control) (C) or subjected to cold hardening (CH), preculture with osmotic agents (Prec), and cryoprotection, with (+LN) or without (−LN) subsequent immersion in liquid nitrogen, following the protocols A and S, which differ in the preconditioning and cryoprotective treatments used (sucrose and PGD I versus sorbitol and DMSO, respectively). Assessments made **(A)** three, **(B)** six and **(C)** nine weeks after treatment. Different letters indicate significant differences with a significance level of 0.05.

**Figure 5:**
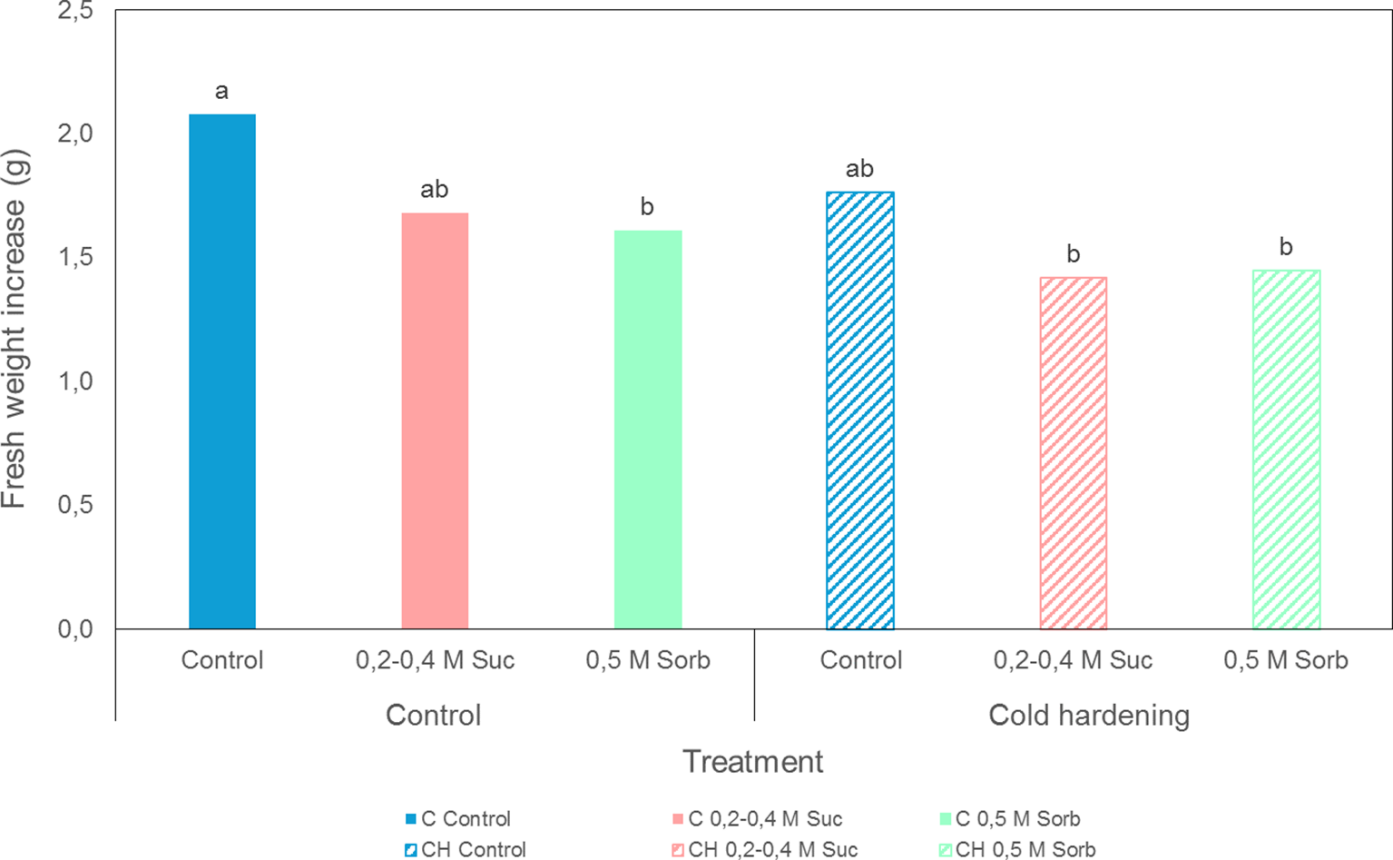
Fresh weight increase of embryogenic cultures of *A. pinsapo* untreated (control) (C) or subjected to cold hardening (CH), and preculture with osmotic agents following the protocols A and S. Assessments made three weeks after treatment. Different letters indicate significant differences with a significance level of 0.05.

Preconditioning treatments, i.e. cold hardening and preculture with osmotic agents, did not affect survival (**Figure 4**), although some modifications could be observed in cultures proliferation capacity (**Figure 5**). In general, samples precultured on culture media containing high concentrations of sucrose or sorbitol presented a yellowish-whitish color compared to control, non-precultured cultures. Furthermore, calli incubated in culture medium with 0.5 M sorbitol showed a viscous consistency. Cold hardening gave rise to harder calli, exhibiting a more compact structure. Statistical analysis revealed a significant effect of cold hardening and preculture on fresh weight increase three weeks after treatments application and transference to standard maintenance conditions (**Supplementary Table S1**). Overall, a decline in the proliferation capacity could be observed after both types of preconditioning treatments.

**Figure 6:**
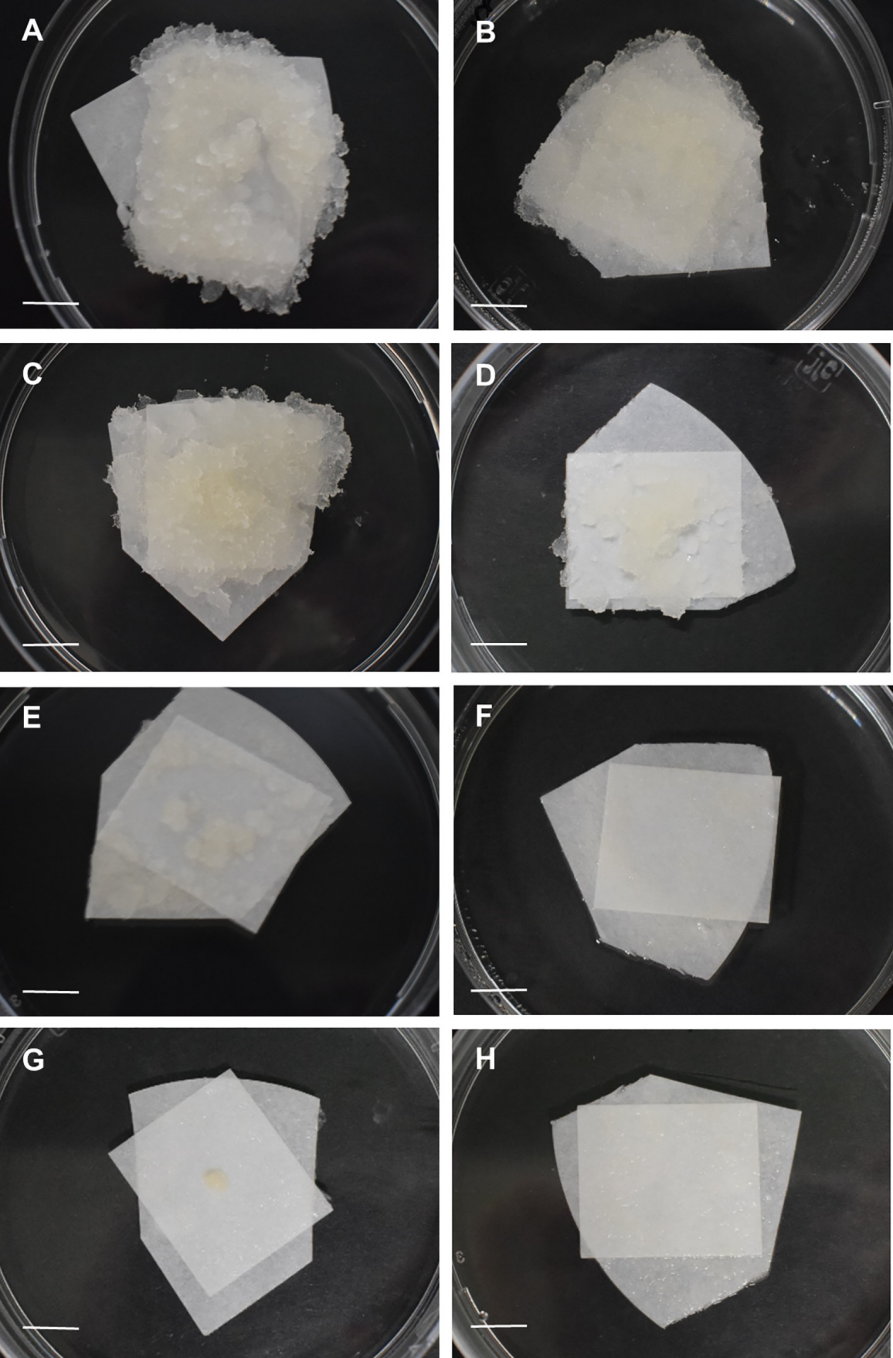
General aspect of embryogenic samples of Spanish fir three weeks after cryopreservation using the slow-cooling method: **(A)** uncooled samples treated following the protocol A (C -LNA), **(B)** uncooled samples treated following the protocol S (C -LNS), **(C)** cold-hardened samples treated following the protocol A (CH -LNA), **(D)** cold-hardened samples treated following the protocol S (CH -LNS), **(E)** uncooled samples cryopreserved following the protocol A (C +LNA), **(F)** uncooled samples cryopreserved following the protocol S (C +LNS), **(G)** cold-hardened samples cryopreserved following the protocol A (CH +LNA), **(H)** cold-hardened samples cryopreserved following the protocol S (CH +LNS). Bar = 2 cm.

Spanish fir embryogenic cultures tolerated well cryoprotection with DMSO (-LNS) or the PGD I solution (-LNA), as no effect on cultures survival were observed in explants subjected to these treatments (**Figure 4**). Both, cold-preconditioned and uncooled calli, actively regrew after application of the cryoprotective treatments. Recovered cultures (**Figures 6A–D**) exhibited a similar aspect to that of control, non-treated calli.

After freezing in liquid nitrogen, first signs of cultures regrowth could be observed one week after thawing, in uncooled samples cryopreserved following the protocol A. However, in cold-hardened calli, new proliferation could not be appreciated up to two weeks later, i.e., three weeks after rewarming. The morphology of tissues arising from cryopreserved explants (+LN) (**Figures 6E–H**) was similar to that of control callus, not subjected to any cryopreservation treatment. The results obtained revealed a significant influence of the protocol on cryopreservation success, with higher survival rates achieved following the procedure A (+LNA) compared to the procedure S (+LNS) (**Figure 4**; **Supplementary Table S2**). Three recultures after thawing, survival rates following the protocol A (+LNA) ranged from 92.31% in uncooled explants to 100% in cold-hardened calli, while in samples cryopreserved following the protocol S recovery rates ranged from 18.18 to 50%, in cold-hardened and uncooled explants, respectively (+LNS).

Overall, a significant influence of cold hardening on explants survival was not found, although six weeks after cryopreservation, three-way log-linear analysis revealed a significant effect of the cold treatment, the protocol, and the interaction between both factors on the recovery of frozen samples (**Figure 4**; **Supplementary Table S2**). Incubation at 5°C for two weeks appeared to affect explants response, delaying tissue regrowth after cryopreservation. As shown in **Figure 4**; although similar recovery rates were reached at the end of the experiment independently of the preconditioning treatment, in cold-hardened explants they were achieved some weeks later.

**Figure 7:**
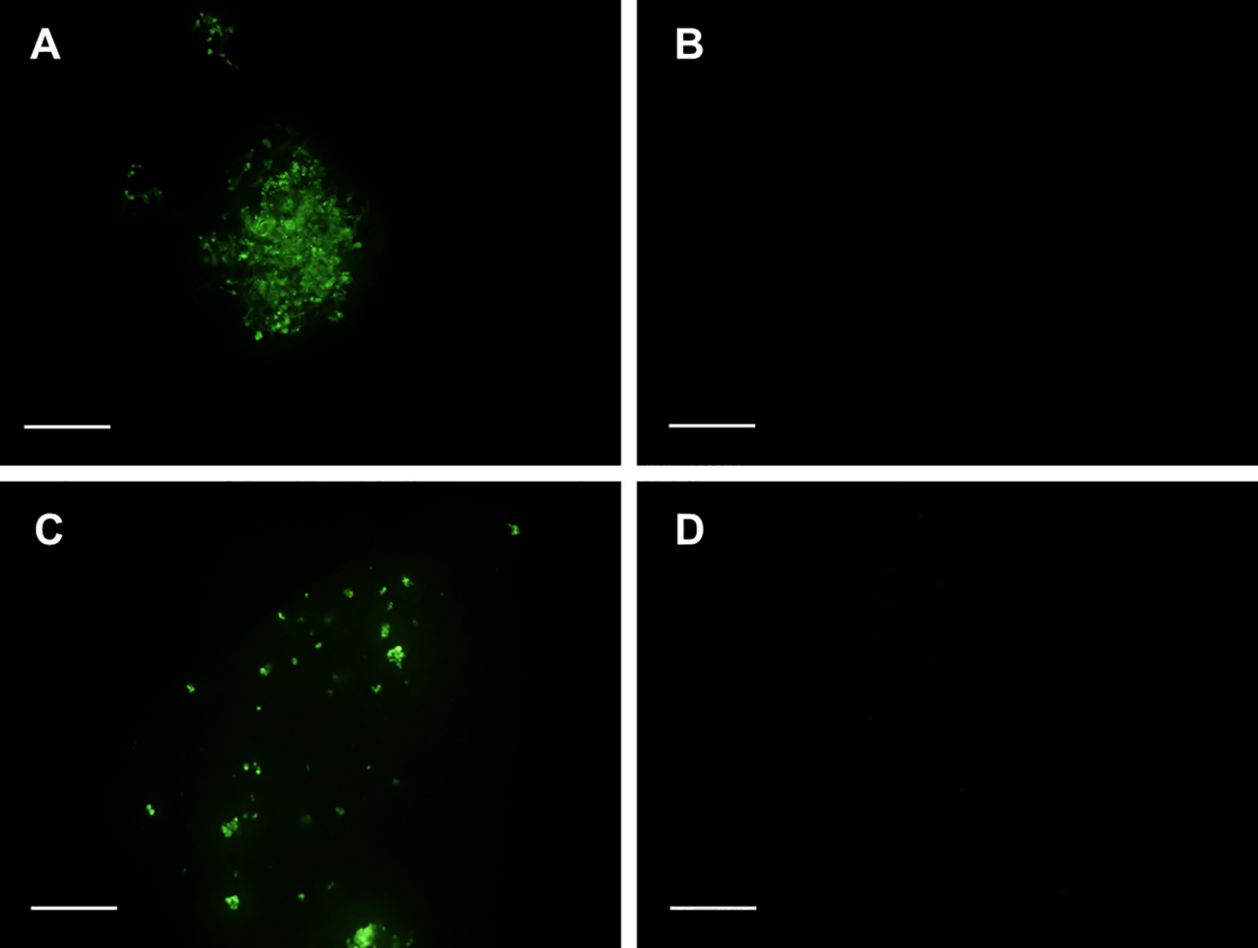
FDA staining of embryogenic samples of Spanish fir three weeks after cryopreservation using the slow-cooling method: **(A)** uncooled samples cryopreserved following the protocol A (C +LNA), **(B)** uncooled samples cryopreserved following the protocol S (C +LNS), **(C)** cold-hardened samples cryopreserved following the protocol A (CH +LNA), **(D)** cold-hardened samples cryopreserved following the protocol S (CH +LNS). Bar = 0.1 mm.

A differential FDA vital staining was observed in samples cryopreserved following the different procedures tested (protocols A and S), with or without a previous cold hardening treatment (**Figure 1**). Three weeks after thawing, green fluorescence was more abundant in uncooled embryogenic tissues frozen after preculture with high sucrose concentrations and cryoprotection with the PGD I solution, as proposed by Aronen et al. (1999) (**Figure 7A**). The proportion of stained cells strongly decreased in samples cryopreserved following the same procedure but previously subjected to a cold treatment (**Figure 7C**). In this case, FDA stained appeared in a disperse way, in few cells or small groups of cells. In samples cryopreserved following the protocol S, FDA vital staining was negligible and it only could be observed when prolonged exposure times were used to make photographs (**Figures 7B, D**).

## 4 Discussion

Somatic embryogenesis has been initiated in a large number of conifer species belonging to the genera *Abies*, *Picea*, *Pinus*, *Pseudotsuga, Taxus* and *Araucaria* (Attree and Fowke, 1993; Lelu-Walter et al., 2013; Salaj et al., 2015; Klimaszewska et al., 2016; Jouini et al., 2023). However, this developmental process has not been previously reported in *A. pinsapo*, a biologically very important species.

Induction of somatic embryogenesis normally requires explants culture on media supplemented with auxin, although cytokinins are also frequently needed (Klimaszewska and Cyr, 2002; von Arnold, 2008). However, in different species of the genus *Abies*, such as *A. fraseri* (Guevin and Kirby, 1997), *A. normanniana*, *A. alba*, *A. cephalonica* (Krajňáková et al., 2008), and *A. nebrodensis* (Jouini et al., 2023), single application of cytokinin was sufficient to induce somatic embryogenesis (Nørgaard and Krogstrup, 1995; Kim et al., 2008; Krajňáková et al., 2008; Salaj et al., 2020). Although diverse cytokinins, such as BA, kinetin, 6-(γ,γ-dimethylallylamino)purine (2iP) and thidiazuron (TDZ), have been tested in different investigations (Nørgaard and Krogstrup, 1991; Kim et al., 2008), BA is the plant growth regulator more frequently used for somatic embryogenesis induction in species of the genus *Abies* (Salajova et al., 1996; Krajňáková et al., 2013; Salaj et al., 2019). In *A. pinsapo*, induction of somatic embryogenesis was achieved in the presence of 5 mg L^-1^ BA, at a 4% induction rate. Similar induction percentages have been reported in other conifer species like *A. balsamea* (3.5%) (Guevin et al., 1994), *A. fraseri* (3.5-18%) (Guevin and Kirby, 1997), *Picea abies* (8%) (von Arnold, 1987), *Abies alba* (0.83–29.4%) (Szczygiel and Kowalczyk, 2001; Krajňáková et al., 2013; Salaj et al., 2020) or *Pinus korainesis* (1.67%) (Gao et al., 2020). Proliferation of embryogenic tissues successfully occurred on culture medium of the same composition supplemented with 500 mg L^-1^ L-glutamine, 1 g L^-1^ casein hydrolysate, and 1 mg L^-1^ BA. Therefore, as found in other *Abies* species (Nørgaard and Krogstrup, 1995; Kim et al., 2008), in *A. pinsapo*, auxins do not play an essential role in somatic embryogenesis, and induction and proliferation of embryogenic cultures can be executed using cytokinins as the sole plant growth regulator.

In this work, successful cryopreservation of Spanish fir embryogenic cultures is also reported. Long-term conservation of plant genetic resources using cryopreservation relies on freezing (Gonzalez-Arnao et al., 2008). Formation of lethal intracellular ice crystals is the main cause of cell damage when exposing biological materials to subzero temperatures (Panis, 1995). Different preconditioning and cryoprotective treatments contribute to protect tissues before freezing. The most common preconditioning treatments include cold hardening and preculture with osmotic agents (Ballesteros et al., 2024). In the present investigation, both types of treatments were applied, with a different influence on the results obtained.

Exposition to low but non-freezing temperatures can induce freezing tolerance, as different processes occur during cold acclimation (Buchanan et al., 2015). Changes in the membrane lipid composition, such as modifications in the proportion of phospholipids, glucocerebrosides or sterol lipids, play a relevant role in the maintenance of the integrity of the plasma membrane, the primary site of injury during freezing (Ramon et al., 2002). Altered lipid-lipid or lipid-protein interactions induced by cold contribute to stabilize the plasma membrane during freeze-thaw cycles. Compatible solutes, such as soluble sugars (like sucrose, glucose, fructose, raffinose, and stachyose) or low molecular weight nitrogen-containing substances (like proline and glycine betaine) also accumulate during cold acclimation. Apart from a role directly related to freezing, compatible solutes also play an important function during osmotic and dehydration stresses, which also may occur during the execution of cryopreservation protocols (Buchanan et al., 2015; Taiz et al., 2015). Proteins involved in very diverse processes, such as phospholipases, dehydrins, antifreeze proteins, stress-related proteins, membrane-trafficking proteins and proteolysis-associated proteins also change in response to low temperatures (Buchanan et al., 2015; Taiz et al., 2015). Most of changes occurring during cold acclimation are mediated by alterations in gene expression. Different cold-responsive pathways appear to be involved in activating cold-induced reactions, thus suggesting complex regulatory systems for genes associated with freezing tolerance (Buchanan et al., 2015).

However, in embryogenic tissues of Spanish fir, cold hardening delayed cultures recovery after freezing in liquid nitrogen. This negative effect, despite the positive molecular processes triggered by cold treatment, could be due to the decreased growth observed in cooled explants. The growth state of cultures critically affects cryopreservation (Bradaï et al., 2023), due to its influence on explants physiological state, which is considered an important factor determining freezing tolerance (Engelmann et al., 2008). As pointed by Bradaï et al. (2017), high metabolic activity and proliferation capacity can play a decisive role on cryopreservation success as it is not only determined by cell survival after thawing, but also by cell ability to proliferate and continue growing later. In this line, in *Pinus caribaea*, Laine et al. (1992) found that only embryogenic cell suspensions rapidly growing could be successfully recovered after liquid nitrogen storage.

Incubation in culture media containing high concentrations of osmotically active agents is a preconditioning treatment frequently used to improve cryotolerance in conifer embryogenic cultures (Häggman et al., 2000). Sugars, such as sucrose, maltose and glucose, and sugar alcohols, such as sorbitol or mannitol, are the most commonly used compounds for pretreatment of conifer embryogenic tissues (Salaj et al., 2010, 2022; Ballesteros et al., 2024). Preculture with osmotic agents has a direct effect decreasing explant´s water content and consequently the amount of intracellular freezable water (Panis and Thinh, 2001), thus avoiding detrimental ice crystal formation during freezing. Besides, mild osmotic stress at which tissues are subjected during preculture with sugars or sugar alcohols increased desiccation and freezing tolerance (Touchell et al., 2002).

In the case of sucrose, it has also been identified as an important signaling molecule regulating metabolism and gene expression (Gupta and Kaur, 2005; Carpentier et al., 2010; Wang et al., 2020). Thus, as pointed by Bradaï et al. (2023), it can increase tolerance to dehydration and cooling by inducing the accumulation of compatible solutes (Suzuki et al., 2006; Pociecha et al., 2009), alterations in fatty acids metabolism (Ramon et al., 2002; Zhu et al., 2006), changes in the proteome (Carpentier et al., 2010; Folgado et al., 2015) or a transient increase of abscisic acid levels (Suzuki et al., 2006). Sorbitol´s positive effects have been attributed to stereochemical arrangements. As suggested by Turner et al. (2001), the stereo-orientation of the hydroxyl groups of sugar alcohols may allow better hydrogen bonding and packing around the membrane bilayer, thus increasing desiccation and freezing tolerance. Pritchard et al. (1986) also reported modifications in cell structure leading to a reduction of the vacuolar volume, due to replacement of the large vacuole by several smaller vesicles.

In embryogenic cultures of Spanish fir, the precultures tested differently affected cultures growth rate. While incubation on proliferation medium supplemented with 0.2 M and 0.4 M sucrose for two successive 24 h-periods did not have a significant detrimental effect, a significant growth reduction could be observed in uncooled, sorbitol-precultured tissues. Besides, despite the short sorbitol pretreatment, variations in cultures morphology could be observed over time.

Cryoprotection also plays an important role in two-step freezing protocols, protecting cells from osmotic damage and ice formation during the cooling steps (Ballesteros et al., 2024). Overall, cryoprotectants appears to act in a multimodal way, with cryoprotection arising from a combination of processes, such as modulation of hydrogen bonding, effects on cell membrane properties, dilution of solute effects and increase of solution viscosity at low temperatures, among others (Murray and Gibson, 2022).

The choice of the cryoprotectant is especially important in the cryopreservation of embryogenic cells, as high water content normally present in this type of tissues, highly increases the risks of intracellular ice crystal formation during freezing (Lambardi et al., 2018). Although DMSO is the cryoprotectant mostly used in conifers (Häggman et al., 2000), different mixtures of penetrating and non-penetrating substances have also been utilized (Cyr et al., 1994; Aronen et al., 1999; Krajňáková et al., 2011; Ozudogru and Lambardi, 2016), as they are often more effective than a single component at the same total osmolarity (Ulrich et al., 1979).

In embryogenic cultures of *A. pinsapo*, the cryoprotectants tested did not have a deleterious effect on cultures recovery. Nevertheless, the best response in terms of survival after freezing was achieved in explants cryopreserved using PGD I. The mixture PGD, including polyethylene glycol, glucose, and DMSO, have been repeatedly utilized in conifers species like *Pinus sylvestris* (Häggman et al., 1998), *Picea abies*, *Pinus taeda* (Gupta et al., 1987), and *Abies cephalonica* hybrids (Aronen et al., 1999). In general, replacement of DMSO by PGD improved the recovery and/or growth of cultures (Häggman et al., 2000). According to Aronen et al. (1999), the other components of the mixture can probably reduce harmful effects of DMSO and retain good growth potential of cultures.

## 5 Conclusions

In this work, somatic embryogenesis has been induced from mature zygotic embryos of *A. pinsapo* cultured on solid MS medium with macroelements at half-strength and supplemented with 5 mg L^-1^ BA. Successful cryopreservation of Spanish fir embryogenic cultures has also been achieved by using the slow-cooling technique. The recovery rates achieved after sucrose preculture and cryoprotection with PGD I (100%) allow secure long-term storage of germplasm of this valuable endangered species. The results obtained allow to establish the bases for the integration of somatic embryogenesis and cryopreservation into *in situ* and *ex situ* strategies for the conservation of *A. pinsapo*.

## Data Availability

The original contributions presented in the study are included in the article/**Supplementary Material**. Further inquiries can be directed to the corresponding author.
