# Supplementary material for: Induction of somatic embryogenesis and cryopreservation of Abies pinsapo Boiss
Source: Front Plant Sci. 2025 Jan 29;16:1535113. doi: 10.3389/fpls.2025.1535113 (PMC11813934; doi:10.3389/fpls.2025.1535113)
Supplement: Supplementary file 1 [file Table1.docx]

**Table S1.** Significance by two-way ANOVA of single and combined effects of cold hardening and preculture with osmotic agents for fresh weight increase of *A. pinsapo* embryogenic cultures, three weeks after treatments application and transference to standard proliferation conditions.

| **Predictor variable** | **Fresh weight increase (g)** |
| --- | --- |
| Cold hardening | **0.006** |
| Preculture | **< 0.001** |
| Cold hardening x Preculture | 0.772 |

Significant *P* values are in bold (P < 0.05).
