# Supplementary material for: Induction of somatic embryogenesis and cryopreservation of Abies pinsapo Boiss
Source: Front Plant Sci. 2025 Jan 29;16:1535113. doi: 10.3389/fpls.2025.1535113 (PMC11813934; doi:10.3389/fpls.2025.1535113)
Supplement: Supplementary file 2 [file Table2.docx]

**Table S2.** Significance by three-way log-linear analysis of single and combined effects of the cryopreservation protocol and cold hardening for recovery rate of cryopreserved (+LN) embryogenic cultures of *A. pinsapo*, three, six and nine weeks after thawing.

| **Predictor variable** | **Recovery (%)** | | |
| --- | --- | --- | --- |
|  | **3 weeks** | **6 weeks** | **9 weeks** |
| Protocol | **0.0002** | **0.0002** | **<0.0001** |
| Cold hardening | 0.1774 | **0.0075** | 0.1618 |
| Protocol x Cold hardening | 0.9987 | **0.0212** | 0.1325 |

Significant *P* values are in bold (P < 0.05).
